# Is home‐based HIV testing universally acceptable? Findings from a case–control study nested within the HPTN 071 (PopART) trial

**DOI:** 10.1111/tmi.13055

**Published:** 2018-04-16

**Authors:** K. Sabapathy, C. Mulubwa, H. Mathema, C. Mubekapi‐Musadaidzwa, A. Schaap, G. Hoddinott, J. Hargreaves, S. Floyd, H. Ayles, R. Hayes, the HPTN 071 (PopART) Study Team

**Affiliations:** ^1^ London School of Hygiene and Tropical Medicine London UK; ^2^ Zambia AIDS Related TB Project Lusaka Zambia; ^3^ Desmond Tutu TB Centre Department of Paediatric and Child Health Stellenbosch University; ^4^ Present address: Division of Public Health Surveillance and Response National Institute for Communicable Diseases National Health Laboratory Service South Africa

**Keywords:** home‐based HIV testing, universal test and treat, case–control study, sub‐Saharan Africa, dépistage du VIH à domicile, dépistage et traitement universels, étude cas‐témoins, Afrique subsaharienne

## Abstract

**Objective:**

The HPTN 071 (PopART) trial is examining the impact of a package including universal testing and treatment on community‐level HIV incidence in Zambia and South Africa. We conducted a nested case–control study to examine factors associated with acceptance of home‐based HIV testing and counselling (HB‐HTC) delivered by community HIV‐care providers (CHiPs) in PopART intervention communities.

**Methods:**

Of 295 447 individuals who were offered testing, random samples of individuals who declined HB‐HTC (cases) and accepted HB‐HTC (controls), stratified by gender and community, were selected. Odds ratios comparing cases and controls were estimated using multivariable logistic regression.

**Results:**

Data from 642 participants (313 cases, 329 controls) were analysed. There were no differences between cases and controls by demographic or behavioural characteristics including age, marital or socio‐economic position. Participants who felt they could be open with CHiPs (AOR: 0.46, 95% CI: 0.30–0.71, *P* < 0.001); self‐reported as not previously tested (AOR: 0.64; 95% CI: 0.43–0.95, *P* = 0.03); considered HTC at home to be convenient (AOR: 0.38, 95% CI: 0.27–0.54, *P* = 0.001); knowing others who had accepted HB‐HTC from the CHiPs (AOR: 0.49, 95% CI: 0.31–0.77, *P* = 0.002); or were motivated to get treatment without delay (AOR: 0.60, 95% CI: 0.43–0.85, *P* = 0.004) were less likely to decline the offer of HB‐HCT. Those who self‐reported high‐risk sexual behaviour were also less likely to decline HB‐HCT (AOR: 0.61, 95% CI: 0.39–0.93, *P* = 0.02). Having stigmatising attitudes about HB‐HTC was not an important barrier to HB‐HCT uptake. Men who reported fear of HIV were more likely to decline HB‐HCT (AOR: 2.68, 95% CI: 1.33–5.38, *P* = 0.005).

**Conclusion:**

Acceptance of HB‐HTC was associated with lack of previous HIV testing, positive attitudes about HIV services/treatment and perception of high sexual risk. Uptake of HB‐HCT among those offered it was similar across a range of demographic and behavioural subgroups suggesting it was ‘universally’ acceptable.

## Abstract (fr)

**Objectif:**

L'essai HPTN 071 (PopART) examine l'impact d'un élément d'ensemble comprenant le dépistage et le traitement universels sur l'incidence du VIH au niveau communautaire en Zambie et en Afrique du Sud. Nous avons mené une étude cas‐témoins imbriquée pour examiner les facteurs associés à l'acceptation du conseil et du dépistage du VIH à domicile (HB‐HTC) fournis par les prestataires communautaires de soins VIH (CHiPs) dans les communautés d'intervention PopART.

**Méthodes:**

Des 295.447 personnes à qui le dépistage a été offert, des échantillons aléatoires de personnes ayant refusé (cas) et ayant accepté le HB‐HTC (témoins), stratifiés selon le sexe et la communauté, ont été sélectionnés. Les rapports de cotes (odds ratios) comparant les cas et les témoins ont été estimés en utilisant une régression logistique multivariée.

**Résultats:**

Les données de 642 participants (313 cas, 329 témoins) ont été analysées. Il n'y avait aucune différence entre les cas et les témoins selon les caractéristiques démographiques ou comportementales, y compris l’âge, la situation conjugale ou socioéconomique. Les participants estimant qu'ils pourraient être ouverts avec CHiPs (AOR: 0,46; IC95%: 0,30‐0,71; *p* <0.001), auto‐déclarant n'avoir pas déjà été testés (AOR: 0,64; IC95%: 0,43‐0,95; *p* = 0,03), considérant que le HB‐HTC était pratique (AOR: 0,38; IC95%: 0,27‐0,54; *p* = 0,001), connaissant d'autres personnes qui avaient accepté le HB‐HTC offert par les CHiPs (AOR: 0,49; IC95%: 0,31‐0,77; *p* = 0,002) ou étant motivés pour obtenir un traitement sans délai (AOR: 0,60; IC95%: 0,43‐0,85; *p* = 0,004), étaient moins susceptibles de refuser l'offre de HB‐HCT. Ceux qui déclaraient avoir un comportement sexuel à risque élevé étaient également moins susceptibles de refuser le HB‐HCT (AOR: 0,61; IC95%: 0,39‐0,93; *p* = 0,02). Avoir des attitudes stigmatisantes à propos du HB‐HTC n’était pas un obstacle important à son adoption. Les hommes qui ont déclaré craindre le VIH étaient plus susceptibles de refuser le HB‐HCT (AOR: 2,68; IC95%: 1,33‐5,38; *p* = 0,005).

**Conclusion:**

L'acceptation était associée à un manque de dépistage précédent du VIH, à des attitudes positives concernant les services/traitements du VIH et à la perception d'un risque sexuel élevé. Le HB‐HCT était acceptable pour les participants de diverses caractéristiques démographiques et comportementales, ce qui suggère qu'il était «universellement» acceptable.

## Introduction

Great advances have been made in controlling the HIV epidemic over time and especially so in the last decade. HIV incidence worldwide has declined, as have HIV‐related deaths 1. The number of people receiving antiretroviral treatment (ART) has increased to 17 million and coverage has reached unprecedented levels even in high‐prevalence countries 2. To gain further ground, a fast‐track strategy is called for to achieve UNAIDS’ 90‐90‐90 targets – with benefits for individual health and prevention of transmission 1, 3, 4, 5, 6, 7. The feasibility of treatment as prevention for public health benefit, whereby a sufficiently high proportion of those infected with HIV know their status, start ART and become virally suppressed so that transmission and HIV incidence may be reduced to a very low level, is currently being tested by a number of studies 8, 9, 10, 11. The HPTN 071/ Population Effects of Antiretroviral Therapy to Reduce HIV Transmission (PopART) trial is being conducted in 21 communities in Zambia and South Africa (with an average population of >50 000 individuals/community) to examine the impact of universal testing and treatment (UTT) on community‐level HIV incidence [9, 12.]

Despite the progress so far, unless uptake of testing is extensive and inclusive in terms of acceptability to all subsets of the population, the full potential of UTT will not be realised. Home‐based HIV testing and counselling (HB‐HTC) has the potential to increase awareness of HIV status in previously undiagnosed individuals in sub‐Saharan Africa 13, 14, 15, 16. The PopART intervention includes door‐to‐door HB‐HTC with the aim of achieving universal testing. A case–control (CC) study on a randomly selected subset of those who had accepted (controls) and those who had declined HB‐HTC (cases) when offered by Community HIV‐care Providers (CHiPs) was carried out, to examine the acceptability of the PopART HB‐HTC intervention during the first year of delivery. In addition to exploring demographic, lifestyle, health and behavioural characteristics, we explored differences in perceptions between cases and controls about factors that may affect uptake of HB‐HTC. We examined participants’ perceptions of HIV services, advantages and disadvantages of accepting HB‐HTC for them as individuals and enquired about stigmatising attitudes which may affect uptake. By comparing non‐acceptors (cases) and acceptors (controls) of HB‐HTC, we hoped to identify any differences and any excluded subsets of the population so that recommendations could be made to help enable universal knowledge of HIV status be achieved through HB‐HTC.

## Methods

**Figure 1:**
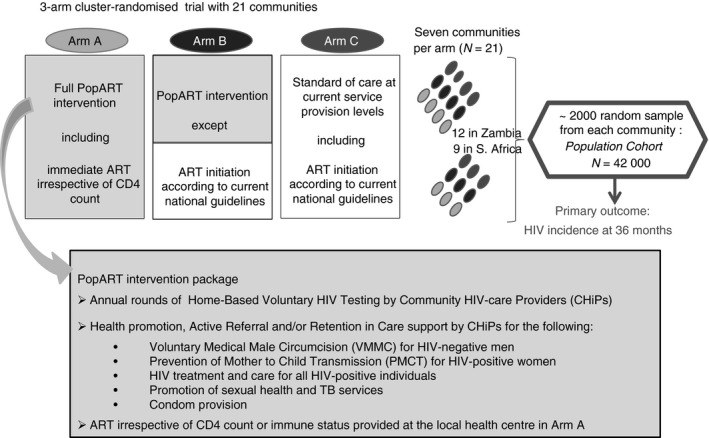
PopART trial schema.

The design of the HPTN 071 (PopART) trial has been described previously 9. Key elements of the trial are shown in Figure 1. Working in pairs, the CHiPs (who were community members employed to work in their communities) were assigned to zones with approximately 500 households to which they delivered the intervention, including HB‐HCT. During the first year of the PopART intervention, 194 795 individuals in Zambia (88 860 men/105 935 women) and 100 652 in South Africa (44 172 men/56 480 women) were offered HB‐HTC by CHiPs in the 14 intervention communities. Of these, 126 208 individuals in Zambia (55 568 men/70 640 women) and 92 375 (40 519 men/51 856 women) in South Africa accepted testing. Individuals who self‐reported HIV‐positive status were not routinely offered testing (and are not included in the above figures).

The nested case–control study was carried out in all the intervention (Arms A and B) communities – eight in Zambia and six in South Africa. The study objectives were to identify differences between non‐acceptors (cases) and acceptors (controls) of HB‐HTC in the first annual round of HB‐HTC in PopART and to identify reasons for non‐acceptance of HB‐HTC.

While delivering the PopART intervention, CHiPs captured the details of all individuals who consented to the intervention offered by CHiPs, irrespective of whether or not they accepted HB‐HTC, on an electronic register 9. From the electronic register which recorded uptake of testing, random samples of non‐acceptors (cases) and acceptors (controls) of HB‐HTC were selected, with a ratio of 1 case:1 control, an equal number of men and women, and an equal number from each community, to have adequate representation of individuals from all the PopART intervention communities and from both genders. It was important to frequency match by gender so that we could ensure we had an adequate sample of men who are often under‐represented in studies of HIV test uptake. An initial random sample in excess of the number needed to be recruited was selected, in anticipation of difficulties in finding participants – due to mobility of community members with frequent change in address.

To be eligible for the case–control study, participants had to be ≥18 years old, able and willing to provide informed consent and have participated in the first year of the PopART intervention. Belonging to the population cohort of the PopART trial (the research cohort in which the trial primary outcome will be measured after 3 years of annual follow‐up) (Figure 1), or to a separate PopART case–control study, was exclusion criteria to avoid research fatigue among study participants. Already being known to be HIV‐infected at the time of the initial CHiP visit was also an exclusion criterion as participants who self‐reported HIV‐positive status were not offered HB‐HTC. HB‐HTC acceptance/non‐acceptance was defined based on whether a community member had accepted/not accepted HB‐HTC offered by CHiPs at the time of random selection in January (Zambia) and March (South Africa) 2015 – representing one year since the start of the intervention in each country.

Verbal permission to allow research staff to approach participants was obtained by the CHiP staff who had provided the intervention to individual community members. Written informed consent for study participation was then obtained by study research assistants (RAs). RAs conducted surveys using standardised questionnaires administered electronically. Questionnaire themes were informed by current evidence in the literature or anecdotal local information on factors that may influence uptake of HIV testing. RAs were kept unaware of participants’ case or control status. In the questionnaire, the question about whether the individual had accepted or declined HB‐HTC was asked at the end of the interview to minimise interviewer bias.

While monitoring data as part of routine quality assurance, the study team uncovered some irregularities in data collection in South Africa. In‐depth internal and independent investigations followed, with oversight from the relevant ethical and regulatory bodies responsible for the study. Consequently, data from one community were not used due to concerns about substantive data irregularities, while in the remaining five communities in South Africa, a rigorous data verification process was undertaken to ensure data integrity. Only participants who could be recontacted and whose data were verified as genuine were retained. The verification process involved confirming the identity of the participant and checking that responses to selected key questions matched responses given during the initial CC visit. No irregularities related to this study were identified in Zambia at any stage.

The final sample size of ~650 participants (1:1 case:control ratio) provided ~80% study power to detect associations with odds ratios of ~1.75 or higher (or ~0.5 or lower), for explanatory variables with 15% prevalence among controls (*α* = 0.05). The age/sex distribution of the final study sample was similar to that of the initial randomly selected sample.

Multivariable logistic regression was used to estimate odds ratios, including community and gender in all models to account for the frequency‐matched sampling strategy. Age category was also included as an a priori potential confounding factor. Additional variables (related to demographic or behavioural characteristics but not opinions or perceptions) for which there was at least weak statistical evidence of association with HB‐HTC acceptance were included as potential confounding variables. Likelihood ratio tests (LRT) were performed to assess the statistical evidence for associations. Evidence of effect modification by gender and country was explored. For variables with three or more response categories and potential for a dose–response relationship, test for trends was performed.

The study was approved by the Ethics Committees of the University of Zambia, Stellenbosch University and the London School of Hygiene and Tropical Medicine.

## Results

**Figure 2:**
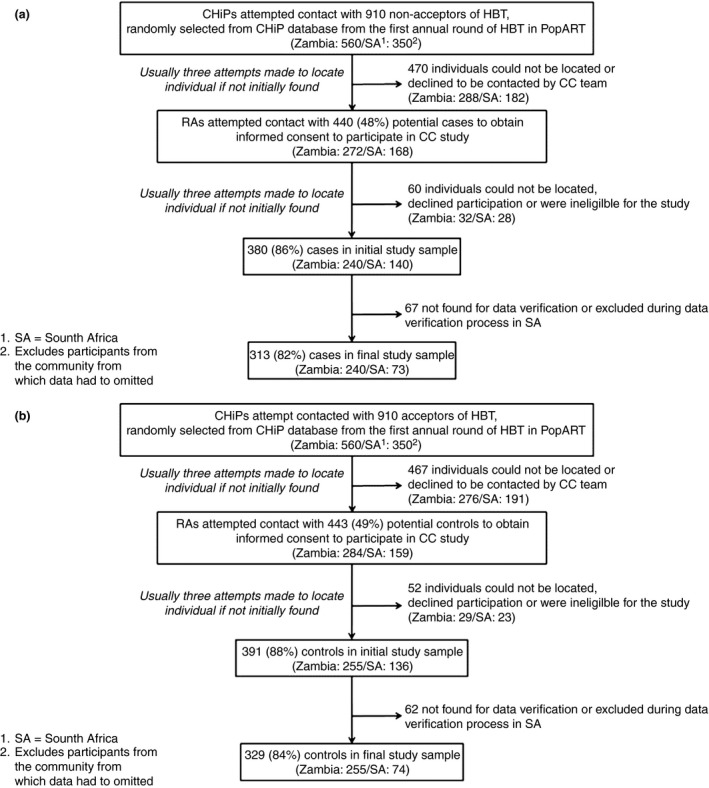
(a) Case (non‐acceptor) selection process and sampling fraction. (b) Control (acceptor) selection process and sampling fraction.

As shown in Figure 2a, of 910 non‐acceptors of HB‐HCT (cases) randomly selected to be contacted by CHiPs, 440 (48%) were found and agreed for their contact information to be passed on to the case–control field research assistants (RAs). Of them, 380 (86%) were consented into the study (Figure 2a). In South Africa, data were verifiable for 73 of the 140 (52%) cases initially recruited there. There were 313 cases in the final study sample. The proportions recruited among potential controls were similar as shown in Figure 2b with 329 controls in the final sample. Data from 642 participants were included in the final analysis – 77% (495) from eight communities in Zambia and 23% (147) from five communities in South Africa (Table 1).

**Table 1:** Demographic and household conditions, and lifestyle, behavioural and health characteristics of cases and controls

	Cases (non‐acceptors) *n* (%)	Controls (acceptors) *n* (%)	Odds ratio^b^	LRT^c^ *P*‐value, 95% confidence interval	Adjusted odds ratio^d^	LRT^c^ *P*‐value, 95% confidence interval
Total	313	329				
Gender						
Male	153 (49)	150 (46)				
Female	160 (51)	179 (54)				
Demographic characteristics						
Age category						*P*_*trend*_ a *0.57*
18–24 years	95 (30)	110 (33)	1	*0.16*	1	*0.34*
25–34 years	79 (25)	98 (30)	0.93	0.62–1.14	0.87	0.57–1.32
35–44 years	73 (23)	54 (16)	1.55	0.98–2.45	1.35	0.84–2.15
≥45 years	66 (21)	67 (20)	1.17	0.74–1.83	1.00	0.63–1.59
Marital status						
Never married	104 (33)	106 (32)	1	*0.63*	1	*0.56*
Currently married	162 (52)	178 (54)	0.89	0.62–1.27	0.78	0.50–1.23
Previously married^e^	47 (15)	45 (14)	1.08	0.65–1.81	0.85	0.46–1.58
Educational attainment						*P*_*trend*_ a *0.21*
Primary (Grade 0–7)	86 (27)	94 (29)	1	*0.81*	1	*0.62*
Junior secondary (Grade 8–9)	72 (23)	81 (25)	0.99	0.64–1.55	1.13	0.71–1.80
Senior secondary (Grade 10–12)	115 (37)	114 (35)	1.12	0.74–1.71	1.32	0.83–2.10
Higher education	40 (13)	40 (12)	1.28	0.72–2.29	1.38	0.75–2.51
Employment						
None	165 (53)	186 (57)	1	*0.36*	1	*0.52*
Casual/seasonal/occasional	43 (14)	44 (13)	1. 00	0.61–1.66	0.97	0.57–1.64
Self employed	49 (16)	37 (11)	1.56	0.93–2.61	1.46	0.85–2.49
Formal wage	56 (18)	62 (19)	0.97	0.63–1.51	1.00	0.63–1.60
Household conditions						
SES (PCA^f^ of HH factors and assets^g^)						
Lower	152 (49)	170 (52)	1	*0.10*	1	*0.15*
Higher	161 (51)	159 (48)	1.36	0.94–1.96	1.31	0.90–1.89
Number of other HH members present when CHiP offered HBT						*P*_*trend*_ a *0.47*
0	113 (37)	122 (38)	1	*0.90*	1	*0.73*
1	83 (27)	87 (27)	0.93	0.62–1.42	0.98	0.64–1.49
≥2	107 (35)	112 (35)	0.91	0.60–1.37	0.85	0.56–1.30
Was partner present when participant offered CHiP HBT?						
N	237 (78)	249 (78)	1	*0.53*	1	*0.47*
Y	66 (22)	72 (22)	0.87	0.56–1.34	0.84	0.52–1.35
Lifestyle, behavioural and health factors Years lived in the community						
≤3	33 (11)	63 (19)	**1**	***0.002***	**1**	***0.003***
≥4	278 (89)	261 (81)	**2.09**	**1.31–3.32**	**2.01**	**1.25–3.22**
Any nights spent away from home in last 3 m						
N	159 (58)	155 (52)	1	*0.19*	1	*0.17*
Y	117 (42)	144 (48)	0.79	0.55–1.13	0.77	0.54–1.19
Number of partners in last 12 m						
0	64 (23)	70 (23)	1	*0.87*	1	*0.79*
1	185 (65)	204 (67)	0.92	0.61–1.38	0.94	0.62–1.43
≥2	35 (12)	31 (10)	1.09	0.59–2.01	1.15	0.61–1.43
Audit score						
Audit score ≤7	242 (77)	260 (79)	1	*0.74*	1	*0.56*
Audit score ≥8	71 (23)	69 (21)	1.07	0.71–1.61	1.13	0.75–1.72
Unwell in last 12 m						
N	208 (67)	224 (68)	1	*0.74*	1	*0.59*
Y	104 (33)	105 (32)	1.06	0.75–1.50	1. 10	0.77–1.58
Any form of violence (verbal/physical/sexual) from any partner in last 12 m (among women)						
No	119 (74)	130 (73)	1	*0.82*	1	*0.90*
At least once	41 (26)	49 (27)	0.94	0.56–1.57	0.98	0.57–1.67

a *P*‐value for test for trend are in italics.

b *A priori* adjusted for gender and community to reflect sampling strategy.

c Likelihood ratio test.

d Multivariable model including gender, community, age category and years lived in the community.

e Previously married = separated/divorced/widowed.

f Principal components analysis.

g HH factors detailed house structure, water, sanitation, electricity and cooking fuel used; assets listed were as follows: working cell phone, bicycle, motorcycle or scooter, car/bakkie, electricity to house, television set, fridge/freezer, radio, computer/laptop, CD or MP3 player, stereo/cassette/other music player, ‘none of the above’.

Bold font indicates findings which are statistically significant but could be shown in normal font if preferred.

### Demographic and household conditions and lifestyle, behavioural and health characteristics

Cases and controls were well balanced by trial arm, community and gender, reflecting the sampling strategy of the study. Participants were distributed fairly evenly across age categories with slightly higher proportions in younger age groups (Table 1). The median age among cases was 32y (IQR: 23–43) and 30y (IQR: 22–40) among controls. The majority of cases and controls were married. While the proportion of participants with higher education was relatively low (12–13%), most had had secondary school education. Most participants were unemployed.

Cases and controls were similarly distributed across almost all the characteristics examined. There were no differences by ethnicity or religion, nor in household conditions, sexual behaviour or health status (including mental health measured by WHO validated Self‐Reported Questionnaire 9, circumcision status and history of pregnancies) (Table 1 and from data not shown).

However, participants who had lived in the community for more than 3 years had twice the odds of declining HB‐HCT than those who had been resident for <3 years (adjusted odds ratio (AOR):2.01,95% confidence interval(95% CI):1.25–3.22, *P* = 0.003).

Neither the number of other household members who were present when HB‐HTC was offered to the household, nor the presence of the participant's partner, were associated with acceptance of HB‐HCT (Table 1).

### Perceptions of HIV services affecting uptake of HB‐HTC

As shown in Table 2, most participants did not know the CHiP prior to the PopART home visit, and there was no association with uptake of HB‐HTC. The majority had faith in the confidentiality of services provided by CHiPs, and there was no difference between cases and controls. However, when asked about whether they could talk openly to the CHiPs, participants who ‘strongly agreed’ that they were comfortable talking openly to CHiPs (who provided HB‐HTC) were less likely to have declined HB‐HTC compared to those who ‘strongly disagreed/disagreed’ (AOR: 0.34, 95% CI: 0.12–0.91, *P* = 0.03). There is evidence of a trend suggesting that the more strongly a participant agreed that they could talk to the CHiP openly, the less likely they were to have declined HB‐HCT (test for trend *P* = 0.003). Also, the more strongly participants agreed that providing treatment widely could reduce incidence of new infections, the less likely they were to have declined HB‐HCT (test for trend *P* = 0.03).

**Table 2:** Participants’ perceptions of HIV service factors affecting uptake of testing

	Cases (non‐acceptors) *n* (%)	Controls (acceptors) *n* (%)	Odds ratio^b^	LRT^c^ *P*‐value, 95% confidence interval	Adjusted odds ratio^d^	LRT^c^ *P*‐value, 95% confidence interval
HIV service factors affecting uptake of testing						
Was the CHiP known to the participant prior to offer of HBT?						
N	265 (85)	272 (83)	1	*0.61*	1	*0.49*
Y	48 (15)	57 (17)	0.88	0.56–1.40	0.85	0.54–1.35
Do you think confidentiality will be maintained by the CHiP?^e^						*p*_*trend*_ a *0.16*
Strongly disagree/disagree	15 (5)	18 (5)	1	*0.10*	1	*0.16*
Agree	98 (31)	82 (25)	1.49	0.69–3.22	1.42	0.65–3.10
Strongly agree	200 (64)	229 (70)	0.95	0.46–2.00	0.91	0.43–1.94
Was the CHiP someone you could talk to openly?^e^						*p*_*trend*_ a *0.003*
Strongly disagree/disagree	12 (4)	7 (2)	1	*0.002*	1	*0.001*
Agree	92 (29)	70 (21)	0.81	0.29–2.24	0.70	0.25–1.94
Strongly agree	209 (67)	252 (77)	0.40	0.15–1.07	**0.34**	**0.12–0.91**
Providing treatment for as many HIV infected people as possible can help reduce new HIV infections happening in your community^e^						*p*_*trend*_ a *0.03*
Strongly disagree	19 (6)	21 (6)	1	*0.09*	1	*0.04*
Disagree	46 (15)	31 (9)	1.54	0.70–3.38	1.63	0.73–3.65
Agree	91 (29)	89 (27)	1.14	0.55–2.35	1.11	0.54–2.31
Strongly agree	156 (50)	188 (57)	0.82	0.42–1.61	0.78	0.39–1.52
Group counselling for HH members (including offer of HIV test) in the home is acceptable e						*p*_*trend*_ a *0.93*
Strongly disagree	41 (13)	43 (13)	1	*0.80*	1	*0.80*
Disagree	31 (10)	34 (10)	1.02	0.53–1.98	0.97	0.50–1.93
Agree	86 (28)	83 (25)	1.20	0.68–2.13	1.21	0.68–2.16
Strongly agree	154 (49)	169 (51)	0.97	0.58–1.61	0.98	0.58–1.63

a *P*‐value for test for trend are in italics.

b *A priori* adjusted for gender and community to reflect sampling strategy.

c Likelihood ratio test.

d Multivariable model including gender, community, age category and years lived in the community.

e There were very few responses in the ‘strongly disagree’ and ‘disagree’ categories for these questions, and responses are therefore grouped as shown to be more meaningful/increase power.

Bold font indicates findings which are statistically significant but could be shown in normal font if preferred.

### Perceived advantages, disadvantages of HB‐HTC

When non‐acceptors and acceptors were asked (identical) standardised questions about factors that encourage HIV testing (regardless of whether they actually did), further associations emerged. Individuals who reported never previously testing for HIV were less likely to have declined HB‐HTC (AOR: 0.64, 95% CI: 0.43–0.95, *P* = 0.03). Similarly, those who knew someone who had had an HIV test with the CHiPs (AOR: 0.49, 95% CI: 0.31–0.77, *P* = 0.002); thought they could get treatment without delay if HIV positive (AOR: 0.60, 95% CI: 0.43–0.85, *P* = 0.004); accepted the CHiP advice that it was good to have an HIV test (AOR: 0.33, 95% CI: 0.23–0.48, *P* < 0.001); and considered testing at home as convenient (AOR: 0.38, 95% CI: 0.27–0.54, *P* < 0.001) – were less likely to have declined HB‐HTC. Participants who indicated that their sexual behaviour put them at risk of HIV (as a reason to test) were also less likely to have declined HB‐HTC (AOR: 0.61, 95% CI: 0.39–0.93, *P* = 0.02).

When exploring reasons against accepting HB‐HTC, those who reported confidence in being HIV negative (so there was no need to test) (AOR: 1.61, 95% CI: 1.04–2.51, *P* = 0.03) and reluctance to test again after recent testing (the definition of recency was not specified and left to the interpretation of the participant) (AOR: 1.69, 95% CI: 1.08–2.67, *P* = 0.02) were more likely to decline HB‐HCT.

In contrast, other factors such as thinking that HIV is common or concerns about confidentiality of HIV testing in the household, that might have influenced uptake of testing, were not found to be associated with acceptance (Table 3). There were no important differences between cases and controls in stigmatising attitudes that may affect uptake of HB‐HTC (Table 3).

**Table 3:** Participants’ perceptions of advantages and disadvantages of accepting of HB‐HTC

	Cases (non‐acceptors) *n* (%)	Controls (acceptors) *n* (%)	Odds ratio^b^	LRT^c^ *P*‐value, 95% confidence interval	Adjusted odds ratio^d^	LRT^c^ *P*‐value, 95% confidence interval
Individual level factors encouraging testing						
When offered a test by the PopART CHiP, did any of the following encourage you towards having an HIV test?						
I have never had an HIV test and wanted to learn my status						
N	247 (79)	237 (72)	**1**	***0.03***	**1**	***0.03***
Y	65 (21)	92 (28)	**0.65**	**0.44–0.96**	**0.64**	**0.43–0.95**
HIV is common in this community so I thought I should test to check my status						
N	217 (70)	223 (68)	1	*0.46*	1	*0.37*
Y	95 (30)	106 (32)	0.87	0.60–1.27	0.84	0.57–1.23
Convenience of having an HIV test at home encouraged me to test						
N	164 (53)	104 (32)	**1**	***<0.001***	**1**	***0.001***
Y	148 (47)	225 (68)	**0.39**	**0.28–0.55**	**0.38**	**0.27–0.54**
Many people I know had tested with a CHiP so I wanted to as well						
N	263 (84)	246 (75)	**1**	***0.001***	**1**	***0.002***
Y	49 (16)	83 (25)	**0.49**	**0.32–0.77**	**0.49**	**0.31–0.77**
Accepted CHiP advice that it was a good idea to test						
N	154 (49)	89 (27)	**1**	***<0.001***	**1**	***<0.001***
Y	158 (51)	240 (73)	**0.35**	**0.24–0.49**	**0.33**	**0.23–0.48**
Getting treatment without delay if I tested and was HIV‐positive (encouraged me to test)						
N	149 (48)	119 (36)	**1**	***0.004***	**1**	***0.004***
Y	163 (52)	210 (64)	**0.61**	**0.44–0.86**	**0.60**	**0.43–0.85**
My sexual behaviour has put me at risk of HIV						
N	263 (84)	257 (78)	**1**	***0.02***	**1**	***0.02***
Y	49 (16)	72 (22)	**0.61**	**0.40–0.94**	**0.61**	**0.39–0.93**
Individual level factors discouraging testing						
When offered a test by the PopART CHiP, did any of the following discourage you from having an HIV test?						
I had difficulty with the time it would take ‐ because of my livelihood/job						
N	226 (72)	247 (75)	1	*0.42*	1	*0.52*
Y	86 (28)	82 (25)	1.18	0.79–1.75	1.14	0.76–1.71
I was worried someone would find out I was having an HIV test						
N	305 (98)	313 (95)	1	*0.08*	1	*0.11*
Y	7 (2)	16 (5)	0.45	0.18–1.12	0.48	0.19–1.22
I did not want to find out my HIV status because I was afraid of a positive test result						
N	263 (84)	288 (88)	1	*0.19*	1	*0.09*
Y	49 (16)	41 (12)	1.38	0.85–2.22	1.53	0.94–2.50
I was confident I was HIV‐negative and didn't need to test						
N	242 (78)	274 (83)	**1**	***0.05***	**1**	***0.03***
Y	70 (22)	55 (17)	**1.53**	**1.00–2.34**	**1.61**	**1.04–2.51**
I already had a test recently and did not want to test again						
N	254 (81)	287 (87)	**1**	***0.03***	**1**	***0.02***
Y	58 (19)	42 (13)	**1.63**	**1.05–2.53**	**1.69**	**1.08–2.67**
I am not ready to find out my HIV status						
N	267 (86)	289 (88)	1	*0.15*	1	0.12
Y	45 (14)	40 (12)	1. 50	0.86–2.64	1.57	0.88–2.77
I just did not want to find out my HIV status (no particular reason)						
N	279 (89)	298 (91)	1	*0.40*	1	0.40
Y	33 (11)	31 (9)	1.32	0.70–2.48	1.32	0.69–2.50
Stigmatising attitudes which may affect uptake of testing						
People are hesitant to take an HIV test due to fear of other people's reaction if the test result is positive for HIV						*P*_*trend*_ a *0.18*
Strongly disagree	69 (22)	72 (22)	1	*0.10*	1	*0.10*
Disagree	54 (17)	49 (15)	1.03	0.57–1.86	0.96	0.52–1.76
Agree	99 (32)	116 (35)	1.28	0.77–2.13	1.20	0.71–2.02
Strongly agree	90 (29)	92 (28)	0.73	0.45–1.18	0.68	0.42–1.12
People sometimes talk badly about people who have had or who are thought to have had an HIV test						*P*_*trend*_ a *0.79*
Strongly disagree	69 (22)	72 (22)	1	*0.78*	1	*0.83*
Disagree	54 (17)	49 (15)	1.10	0.64–1.87	0.99	0.58–1.71
Agree	99 (32)	116 (35)	0.86	0.53–1.39	0.83	0.51–1.35
Strongly agree	90 (29)	92 (28)	1.02	0.62–1.67	0.98	0.59–1.62
People may think that I have been immoral/irresponsible as the reason behind having an HIV test						*P*_*trend*_ a *0.53*
Strongly disagree	129 (41)	146 (44)	1	*0.60*	1	*0.53*
Disagree	69 (22)	77 (23)	0.96	0.62–1.48	0.90	0.57–1.40
Agree	68 (22)	60 (18)	1.34	0.82–2.21	1.33	0.80–2.20
Strongly agree	46 (15)	46 (14)	1.05	0.62–1.79	1.04	0.60–1.79
People receive verbal abuse or insults because of having an HIV test						*P*_*trend*_ a *0.90*
Strongly disagree	43 (14)	44 (13)	1	*0.61*	1	*0.82*
Disagree	73 (23)	84 (26)	1.33	0.85–2.06	1.21	0.77–1.90
Agree	78 (25)	65 (20)	1.03	0.65–1.62	1.02	0.64–1.62
Strongly agree	118 (38)	136 (41)	1.04	0.61–1.77	0.97	0.56–1.69

a *P*‐value for test for trend are in italics.

b A priori adjusted for gender and community to reflect sampling strategy.

c Likelihood ratio test.

d Multivariable model including gender, community, age category and years lived in the community.

Bold font indicates findings which are statistically significant but could be shown in normal font if preferred.

### Differences in association by gender and country

There were few differences observed when stratifying associations by gender and country (Table S1a,b). Men who stated that they feared an HIV‐positive test result were more likely to have declined HB‐HTC (AOR: 2.68, 95% CI: 1.33–5.38, *P* = 0.005), whereas no such association was noted among women (AOR: 0.84, 95% CI: 0.39–1.80, *P* = 0.65) (LRT for interaction with gender *P*‐value = 0.005).

## Discussion

Our study provides evidence from large urban communities that were targeted to receive universal testing (and in Arm A communities, universal treatment as well) (Figure 1). UTT has the potential to influence acceptability and uptake of HIV testing and only one other quantitative study to our knowledge has reported findings on predictors of uptake from a setting providing UTT. This study was on data from a much smaller trial than PopART, set in rural South Africa with 10 clusters and an average population size of approximately 1000 individuals/cluster), and only a few potential factors associated with the uptake of HB‐HTC were described [17.] While there are descriptive studies of acceptors of testing and HB‐HTC, relatively few studies have directly compared acceptors with non‐acceptors of HB‐HTC, and in‐depth quantitative data on reasons to decline are limited 17, 18, 19, 20. HB‐HTC acceptance has been shown to be associated with age (>25 years) and female gender in Kenya 18, and low socio‐economic position in a the setting of a small island in Lake Malawi 19. Other data have shown no association between HB‐HCT uptake and demographic or socio‐economic position 17. Prior knowledge of HIV status (known HIV‐infected or believing oneself to be uninfected based on a previous HIV‐negative test result) and not being ready to find out have been found as reasons to decline HB‐HTC in rural South Africa 20. Others have reported little that is significantly different between those who accepted and those who did not accept HB‐HTC 17.

Our study sample was frequency‐matched by gender and community to ensure adequate representation of those groups. As such, rather than identify whether there were any differences in uptake by gender (other PopART data on uptake of HB‐HCT answer that 21), we were able to explore differences between those who declined and those who accepted HB‐HTC after accounting for gender. We were also able to examine whether associations differed by the gender of the participant.

From our study within 13 large urban communities in Zambia and South Africa, we found that among those who were encountered and offered HB‐HTC, there were no fundamental differences based on demographic, lifestyle, behavioural or health characteristics, between those who accepted (controls) and those who declined HB‐HCT (cases). Our data indicate that there were no specific subsets of the population who were systematically less likely to accept testing, once contacted, suggesting that HB‐HTC has the potential to be universally acceptable to those offered it. Evidence indicates that there are fewer men found at home than women and HB‐HTC providers may therefore encounter less men 17, 21. To achieve universal coverage, innovative means must be explored to ensure everyone in the community (or as high a proportion as possible) is contacted so that they can be offered HB‐HTC 22.

Cases and controls did seem to differ in perceptions held about issues related to HIV and HIV services. Most participants gave favourable responses regarding HIV services, and those who held positive views about the CHiPs were less likely to have declined HB‐HTC. There were several factors that encouraged testing at the individual level. Participants who had not tested for HIV previously were more likely to accept HB‐HTC in contrast to those who had previously tested HIV negative or had tested recently and felt that repeat testing was not warranted.

Participants who declined HB‐HCT were less positive about treatment for HIV than those who accepted. Further, those who declined were more likely to hold the view that they were not at risk of HIV and it was therefore not a reason for them to test. Low‐risk perception as a reason not to test was also observed by Naik *et al* [20.] Yet when we explored self‐reported sexual behaviour of participants, there is no evidence that those who declined HB‐HCT were at lower risk based on the number of partners in the last 12 months (Table 1), number of lifetime partners or age at sexual debut (data not shown).

Other views that might have been assumed to encourage or discourage testing had no association with observed acceptance of HB‐HTC. For example, concerns about confidentiality with testing in the home, or the presence of other household members during delivery of HB‐HTC (including partner), were not associated with acceptance and so these factors did not appear to inhibit testing. Contrary to other studies 20, ‘not feeling ready to find out’ his/her HIV status was not found to be associated with acceptance in our study. Having stigmatising attitudes about HB‐HTC was also not seen to be an important barrier to uptake in our setting.

We found surprisingly few differences between responses given by men and women. However, the data do suggest that among men, fear of an HIV‐positive result was associated with HB‐HTC non‐acceptance.

Further research is needed to explain some study findings, including the association of longer duration lived in the community with non‐acceptance, or the finding that greater mobility is associated with increased likelihood of acceptance in Zambia. Several of the communities studied have been exposed to HTC campaigns in the past. Individuals who have been resident for longer periods may have been tested before and therefore declined HB‐HTC when offered by PopART CHiPs. In contrast, mobility is associated with higher sexual risk 23 and individuals who are mobile may be more inclined to accept HB‐HTC if they feel at risk of HIV. Social science research is being conducted on a subset of the participants from this case–control study and in‐depth interviews may provide more nuanced explanations. There are other components of the trial which are using qualitative methods of research which may help provide more nuanced explanations concerning the effects of mobility and permanence in the community on uptake of HB‐HTC.

Our study had some limitations to consider. To comply with ethical principles and good research practice, only individuals who were encountered and agreed to participate in the PopART intervention, and who were recontacted and provided informed consent for the CC study, could be recruited as participants. Due to high mobility in the study communities, randomly selected individuals from the CHiP database were often difficult to trace, to ask permission for contact by the research team. As such, it may be that the study sample is not fully representative of all community members and our results should be interpreted in the light of this limitation. However, the response rates in cases and controls were very similar (as seen in Figure 2a,b) indicating that any selection bias was likely non‐differential, so that comparisons between cases and controls should be valid.

Finally, in common with most research using self‐reported data, reporting bias is possible. Social desirability may have played a part in the responses given, although we would not expect this to be differential based on whether an individual had accepted HB‐HTC for most themes studied. We also minimised observer bias by keeping research assistants unaware of case–control status of participants until the end of the questionnaire.

However, our study also had several strengths. There was no single or obvious hypothesis being tested, so respondents were unlikely to give responses in order to conform to (or contradict) such a hypothesis. In contrast to much of the existing literature on acceptability of HIV testing, our study is specific to the context of attempting to provide universal testing and at large scale. Further, we directly compared those who accepted HB‐HTC with those who did not to provide evidence of differences rather than simply describing individuals without comparators. Also, by frequency matching our study sample by gender, we ensured an adequate sample of men who are often under‐represented in studies of HIV test uptake despite (or because of) the fact that they are more frequently non‐engagers with HIV services. Finally, the study covered an extensive range of themes. The null findings make an important contribution to identifying which areas may be less important when designing public health information to encourage HB‐HCT.

While firm evidence of causality cannot be inferred from this observational study, our study findings provide opportunities for tailoring services and public health messaging to extend the reach of HB‐HTC, to those who may currently be avoiding it. Our first key recommendation is with regard to decision‐making about testing being based on whether one needs a test. We found that those who had previously tested HIV negative or had tested recently felt that repeat testing was not warranted. Also, participants who indicated that their sexual behaviour put them at risk of HIV were less likely to have declined HB‐HTC. We recommend that service providers reinforce the importance of testing irrespective of self‐held perceptions of risk of HIV, especially where universal knowledge of HIV status is sought. WHO guidelines do not recommend retesting to cover a ‘window‐period’ 24 and it is reasonable not to retest following a test in the last 3 months. However, if there is any potential for ongoing exposure, repeat and ongoing testing (e.g. annually) should be encouraged from a public health point of view. The failure to retest because of a past HIV‐negative result may be complacent, especially in high‐prevalence settings. Data from PopART intervention delivery indicate high acceptability of HB‐HTC provided by CHiPs 25. Data from this study which indicate that those who held positive views about the CHiPs were less likely to have declined HB‐HTC highlight the benefits to be gained by maximising the acceptability of the cadre of staff delivering HB‐HTC, which may help us improve uptake and reach universality. Similarly, promoting the benefits of treatment may have benefits for uptake of testing. Among men, fear of HIV was found to influence test uptake and efforts must be made to understand and mitigate it. We recommend that there should be investment in health promotion which demystifies HIV – through expansion of channels to target men (health promotion aligned with sporting events and activities, or tailored male‐friendly services, for instance).

## Conclusion

This case–control study, which is nested within the largest HIV prevention trial to date, provides valuable insights into the acceptability of HB‐HTC. We found that that there were no differences in uptake of HB‐HTC by demographic and behavioural characteristics suggesting that HB‐HTC has the potential to be universally acceptable – to those who can be contacted and offered it – although to achieve universal coverage, innovative ways to make contact and offer HB‐HTC extensively will be needed. Ideally, a mixture of approaches (including stand‐alone, health facility, community location and home‐based methods) should be made available, so that individuals have a choice and coverage may be maximised. We have identified perceptions and opinions held by community members that could help tailor public health messaging with a view to achieving universal knowledge of HIV status in high‐prevalence settings.

## Supporting information


**Table S1** (a) Factors with effect modification by gender, of association with case/control status. (b) Factors with effect modification by country, of association with case/control status.
