# Supplementary material for: Is home‐based HIV testing universally acceptable? Findings from a case–control study nested within the HPTN 071 (PopART) trial
Source: Trop Med Int Health. 2018 Apr 16;23(6):678–90. doi: 10.1111/tmi.13055 (PMC6001569; doi:10.1111/tmi.13055)
Supplement: Supplementary file 1 — Table S1 (a) Factors with effect modification by gender, of association with case/control status. (b) Factors with effect modification by country, of association with case/control status. [file TMI-23-678-s001.docx]

**Supplementary Table 1a.** Factors with effect modification by gender, of association with case/control status

|  | **Controls (Acceptors) N (%)** | **Cases (Non-acceptors) N (%)** | **Odds Ratio ^1^** | ***LRT ^2^ p-value*, 95 % Confidence Interval** | **Adjusted Odds Ratio^3^** | ***LRT ^2^ p-value,*  95 % Confidence Interval** | **Controls (Acceptors) N (%)** | **Cases (Non-acceptors) N (%)** | **Odds Ratio ^1^** | ***LRT ^2^ p-value*, 95 % Confidence Interval** | **Adjusted Odds Ratio^3^** | ***LRT ^2^ p-value,*  95 % Confidence Interval** |
| --- | --- | --- | --- | --- | --- | --- | --- | --- | --- | --- | --- | --- |
|  | **Men** | | | | | | **Women** | | | | | |
| ***Individual level factors affecting testing*** | | | | | | | | | | | | |
| **I did not want to find out my HIV status because I was afraid of a positive test result** | | | | | | | | | | | *p_effect modification_ ^4^ 0.02* | |
| N | 132 (88) | 119 (78) | **1** | ***0.01*** | **1** | ***0.005*** | 156 (87) | 144 (90) | 1 | *0.47* | 1 | *0.65* |
| Y | 18 (12) | 33 (22) | **2.27** | **1.16-4.44** | **2.68** | **1.33-5.38** | 23 (13) | 16 (10) | 0.76 | 0.36-1.60 | 0.84 | 0.39-1.80 |
| **I just did not want to find out my HIV status (no particular reason)** | | | | | | | | | | | *p_effect modification_ ^4^ 0.05* | |
| N | 131 (86) | 138 (92) | **1** | ***0.03*** | **1** | **0.02** | 71 (97) | 71 (96) | 1 | *0.27* | 1 | *0.20* |
| Y | 21 (14) | 12 (8) | **2.58** | **1.09-6.10** | **2.83** | **1.17-6.86** | 3 (2) | 3 (4) | 0.56 | 0.20-1.59 | 0.50 | 0.17-1.47 |
| **People sometimes talk badly about people who have had or who are thought to have had an HIV test** | | | | | | | | | | | *p_effect modification_ ^4^ 0.02* | |
| Strongly disagree | 32 (21) | 32 (21) | 1 | *0.39* | 1 | *0.43* | 37 (23) | 40 (22) | 1 | *0.05* | 1 | ***0.04*** |
| Disagree | 25 (16) | 20 (13) | 1.06 | 0.47-2.37 | 1.00 | 0.44-2.26 | 29 (18) | 29 (16) | 1.04 | 0.50-2.16 | 0.94 | 0.44-1.98 |
| Agree | 58 (38) | 48 (32) | 1.26 | 0.63-2.53 | 1.24 | 0.61-2.54 | 41 (26) | 68 (38) | 0.54 | 0.27-1.08 | 0.50 | 0.25-1.02 |
| Strongly agree | 37 (24) | 50(33) | 0.72 | 0.34-1.50 | 0.71 | 0.34-1.51 | 53 (33) | 42 (23) | 1.28 | 0.65-2.52 | 1.25 | 0.62-2.52 |

1. A priori adjusted for gender and community to reflect sampling strategy
2. Likelihood ratio tes
3. Multivariable model including gender, community, age category and years lived in the community
4. LRT p-value indicating evidence of effect modification by gender

**Supplementary Table 1b.** Factors with effect modification by country, of association with case/control status

|  | **Controls (Acceptors) N (%)** | **Cases (Non-acceptors) N (%)** | **Odds Ratio ^1^** | ***LRT ^2^ p-value*, 95 % Confidence Interval** | **Adjusted Odds Ratio^3^** | ***LRT ^2^ p-value,*  95 % Confidence Interval** | **Controls (Acceptors) N (%)** | **Cases (Non-acceptors) N (%)** | **Odds Ratio ^1^** | ***LRT ^2^ p-value*, 95 % Confidence Interval** | **Adjusted Odds Ratio^3^** | ***LRT ^2^ p-value,*  95 % Confidence Interval** |
| --- | --- | --- | --- | --- | --- | --- | --- | --- | --- | --- | --- | --- |
|  | **Zambia** | | | | | | **South Africa** | | | | | |
| ***Demographic characteristics*** | | | | | | | | | | | | |
| **Any nights spent away from home in last 3m** | | | | | | | | | | | *p_effect modification_ ^4^ 0.05* | |
| N | 110 (54) | 100 (44) | **1** | ***0.03*** | **1** | ***0.03*** | 49 (67) | 55 (75) | 1 | *0.26* | 1 | *0.49* |
| Y | 93 (46) | 126 (56) | **0.64** | **0.43-0.97** | **0.63** | **0.41-0.96** | 24 (33) | 18 (25) | 1.53 | 0.72-3.29 | 1.33 | 0.59-2.97 |
| ***Individual level factors affecting testing*** | | | | | | | | | | | | |
| **I had difficulty with the time it would take - because of my livelihood/job** | | | | | | | | | | | *p_effect modification_ ^4^ 0.03* | |
| N | 176 (69) | 162 (68) | **1** | ***0.87*** | **1** | ***0.83*** | 7 1 (96) | 3 (4) | 1 | *0.08* | 1 | *0.03* |
| Y | 79 (31) | 77 (32 | **1.04** | **0.68-1.58** | **0.95** | **0.62-1.47** | 64 (88) | 9 (12) | 3.22 | 0.79-1.321 | 4.73 | 1.02-21.98 |

1. A priori adjusted for gender and community to reflect sampling strategy
2. Likelihood ratio test
3. Multivariable model including gender, community, age category and years lived in the community
4. LRT p-value indicating evidence of effect modification by country
